# Enhancement of Cooperation and Reentrant Phase of Prisoner’s Dilemma Game on Signed Networks

**DOI:** 10.3390/e24020144

**Published:** 2022-01-18

**Authors:** Jae Han Choi, Sungmin Lee, Jae Woo Lee

**Affiliations:** 1 Department of Physics, Inha University, Incheon 22212, Korea; powerwin11@gmail.com; 2 R & D Center, PharmCADD Co., Seoul 06180, Korea; 3 Institute of Advanced Computational Sciences, Inha University, Incheon 22212, Korea

**Keywords:** game theory, prisoner’s dilemma game, signed networks, cooperation

## Abstract

We studied the prisoner’s dilemma game as applied to signed networks. In signed networks, there are two types of links: positive and negative. To establish a payoff matrix between players connected with a negative link, we multiplied the payoff matrix between players connected with a positive link by −1. To investigate the effect of negative links on cooperating behavior, we performed simulations for different negative link densities. When the negative link density is low, the density of the cooperator becomes zero because there is an increasing temptation payoff, *b.* Here, parameter *b* is the payoff received by the defector from playing the game with a cooperator. Conversely, when the negative link density is high, the cooperator density becomes almost 1 as *b* increases. This is because players with a negative link will suffer more payoff damage if they do not cooperate with each other. The negative link forces players to cooperate, so cooperating behavior is enhanced.

## 1. Introduction

Game theory has been applied in many areas, such as economics, ecology, politics, and behavioral science. In game theory, the agents are rational, so they adapt to their neighbor’s behavior to maximize their payoff. The most popular model of game theory is the prisoner’s dilemma (PD), where two agents can choose one of two strategies: cooperate or defect. The defector obtains a greater benefit than the cooperator in the PD game [1,2,3,4,5,6,7,8,9,10,11,12]. When two players are cooperating (C), they gain payoff R (a reward for mutual cooperation). Two defectors (D) obtain payoff P (punishment for mutual defection) when they play. When a defector meets a cooperator in the game, the defector obtains payoff T (the temptation to defect), whereas the cooperator obtains the “sucker’s payoff” (S). In the PD game, the payoff satisfies an inequality relation as follows: T>R>P>S. There is an equilibrium strategy (D, D), the so-called Nash equilibrium. In this equilibrium, the two players simultaneously choose the same strategy (D) [1,2,3,4,5,6,7,8,9,10,11,12]. Two other interesting games are the snow drift game (T>R>S>P) and the stag hunt game (R>T>P>S).

In real life and ecological systems, one observes cooperation among selfish players [13,14,15,16,17,18,19]. Spatial structures play vital roles in cooperation during the PD game [15,16,17]. Nowak and May introduced a spatial PD game in a two-dimensional lattice [13]. They observed persistent survival of cooperators and defectors forming spatial chaotic patterns. Szabo and Toke introduced the PD game in a square lattice [14]. The game exhibited a continuous phase transition between absorbing states for the temptation to defect control parameter. Nowak et al. studied fairness in the ultimatum game on the spatial structure [10]. Wang et al. reviewed evolutionary games in multilayer networks [19]. They discussed the promotion of cooperation from the connections of players in a network of networks. Wu et al. reported that cooperation in the PD game was sometimes enhanced and sometimes inhibited in a scale-free network [20]. Rong et al. observed that degree-mixing patterns of complex networks have influenced cooperation and promotion of invasion by defectors in the PD game [21]. Yang et al. studied the dynamic organization of cooperator clusters in a spatial prisoner’s dilemma [22]. They offered a condition for the dominance of cooperation, finding that a phase transition characterized by the emergence of a large spanning cooperator cluster occurs when the initial fraction of cooperators exceeds a certain threshold. Li et al. studied cooperation in spatial prisoner’s dilemma games with a reward mechanism. They found that the reward mechanism is of great benefit to cooperation [23]. Gong et al. introduced reputation-based co-evolutionary prisoner’s dilemma games. They reported that the massive scale of reputation fluctuation helps to enhance the cooperative effect on different network topologies [24]. Szolnoki and Perc observed that conformity enhances network reciprocity in an evolutionary social dilemma [25].

Recently, the signed network has drawn attention by reflecting more realistic interactions, such as friend or foe, activation or inhibition, and so on. There are two types of link in the signed network: positive and negative. Tan and Lu reported evolutionary game dynamics in a signed network [26]. They observed minimization of structural conflicts when players adjusted their choice of alliance. Hiller introduced a simple model of signed network formation, where strong agents extract payoffs from weak enemies [27]. Aref and Wilson considered measuring a partial balance in signed networks [28]. He et al. reported the important role of structural balance in the dynamics of signed networks [29]. They found that cooperation prevails when individuals have a higher probability of adjusting the signs of their relations. Lin et al. considered a game where two stubborn agents compete to maximize the expected number of non-stubborn agents adopting their opinions in the signed network [30]. They observed that a stubborn agent can increase the payoff by selecting a suitable non-stubborn agent to connect to. Sheykhali et al. observed that each agent in a signed network has a mixture of positive and negative links representing friendly or antagonistic interactions and his stubbornness about interactions [31].

In reality, the strength of cooperation is different among communities. Moreover, relationships such as friend/foe or like/dislike are mixed differently among communities. In this study, we investigate how the mixture of positive/negative relationship affects cooperating behavior. To do so, we extend the evolutionary prisoner’s dilemma game to a signed network. We consider a mixed game that consists of the prisoner’s dilemma game in the positive link and the reverse prisoner’s dilemma game in the negative link. In the reverse PD game, two players use the payoff matrix in which minus one is multiplied to elements of payoff matrix in the PD game. This mixed game pretends social behavior uses different strategies when players behave in society. We also report the reentrant phase in the phase diagram. When there are no signed links, the evolutionary spatial dynamics shows a phase transition from a cooperating state to a defecting state at a critical point. When we introduce the signed network, we observe that the cooperating state reappears at the high value of a control parameter. In Section 2, we introduce the model. The mean-field model of the game is considered in Section 3, and the results are discussed. Concluding remarks are provided in Section 4.

## 2. Models

**Figure 1:**
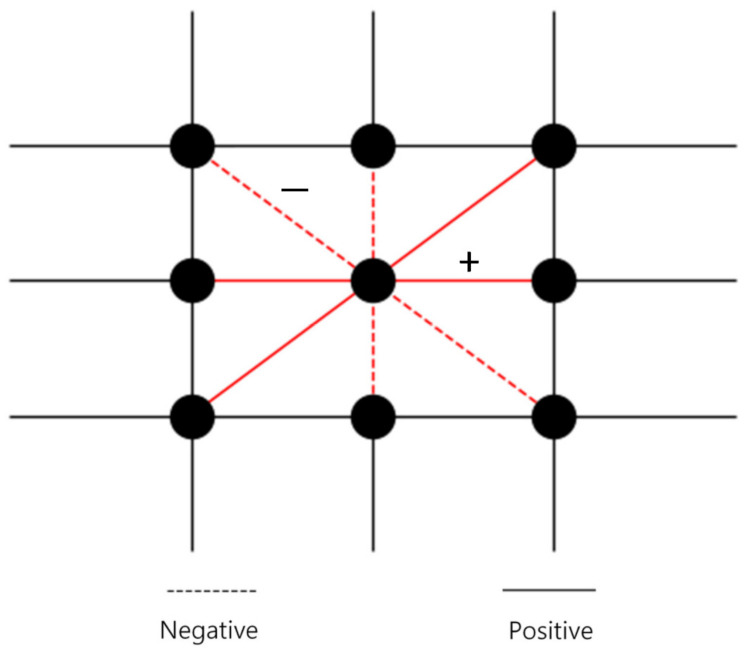
Prisoner’s dilemma game on a signed square lattice with eight neighbors. Two players with a positive link play with the original payoff matrix. However, two players with a negative link play with a different payoff matrix.

We consider a prisoner’s dilemma game on a signed square lattice with Moore’s nearest neighbors (eight neighbors), as shown in Figure 1. We use a 1000×1000 square lattice with a periodic boundary condition in the simulation. Each player can choose one of two strategies: cooperation (C) or defection (D). We have two types of signed links. One type of link is positive. With the positive link, two players play the Nowak and May [3] simple prisoner’s dilemma game shown in Table 1. The other type of link is negative. With the negative link, two players take payoffs that are multiplying minus one to elements of payoff matrix in the PD game; that is, the cooperator obtains a payoff of −1 when he/she plays with a cooperator; however, the defector obtains a payoff of −b when he/she plays with a cooperator, where b>1. Therefore, with the negative link, cooperators outperform defectors when they meet a cooperator. We refer to this game with a negative link as the reverse prisoner’s dilemma game. In it, the players with a positive link receive profits whereas the players with a negative link incur losses. However, when a cooperator or defector plays with the defector, their payoffs are zero. Therefore, they keep their original state and resist the defector. As a result, the players with a positive link take the best strategy with the best profit and the players with a negative link take the best strategy to avoid loss.

To consider the effect of the negative link in the modified prisoner’s dilemma game, we vary the ratio of negative links, defined as:

(1) $$r_{-}=\frac{N_{-}}{N}$$

where N is the total number of links in the system, $N_{-}$ is the total number of negative links, and $N_{+}=N-N_{-}$ is the total number of positive links in the system.

Each player on the lattice has eight nearest neighbors in the system. In each round, we select node i and nearest neighbor j. The player plays according to his/her total payoff and the total payoff of the neighbors. In each round of the game, the players calculate their total payoff as the sum of the payoffs between pairs of nearest neighbors. When the link is positive, the payoff is a positive value. However, if the link is negative, the payoff is a negative value. Let the total payoff of player i be $U_{i}$. The state of the player is updated according to the updating probability as follows:

(2) $$W\left(i\leftarrow j\right)=\frac{1}{1+exp\left(\frac{-\left(U_{j}-U_{i}\right)}{k}\right)}$$

where $U_{j}$ is the total payoff of the randomly selected neighbor, and k is a control parameter that represents probabilistic uncertainty, such as an inverse temperature. We set parameter k=0.04.

## 3. Results and Discussion

**Figure 2:**
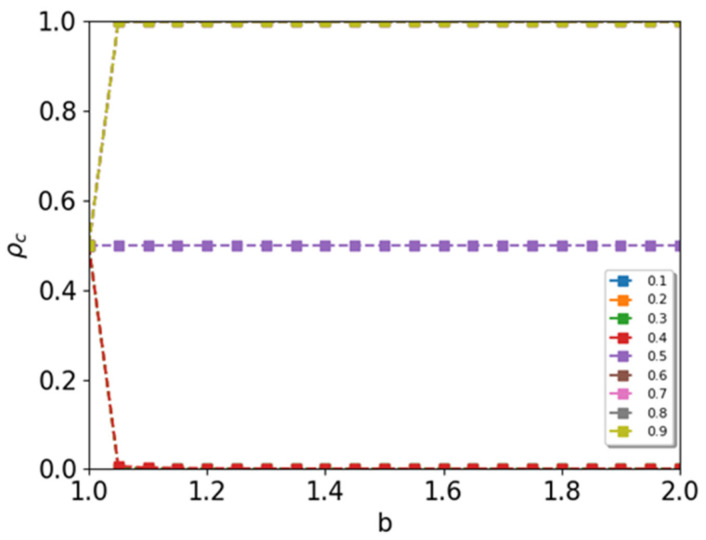
Mean-field results of the order parameter $\rho_{c}\;$in the mixed spatial game on a signed square lattice. Two players with a positive link play with the original payoff matrix of the PD game. However, two players with a negative link play with a different payoff matrix of the reverse PD game.

First, we calculated the mean-field rate equation (see Appendix A). The stationary solution of the mean-field rate equation is numerically obtained as shown in Figure 2. Initially, we start the simulation with the density of cooperators $\rho_{c}=0.5$, then it approaches to zero ($\rho_{c}\to 0$) for $r_{-}<0.5$, and $\rho_{c}\to 1$ for $r_{-}>0.5$. For $r_{-}=0.5$, cooperators and defectors coexist where $\rho_{c}=0.5$. If the ratio of negative links deviates from $r_{-}=0.5$, the density of cooperators in the steady state approaches saturation value zero or 1, as shown in Figure 2.

**Figure 3:**
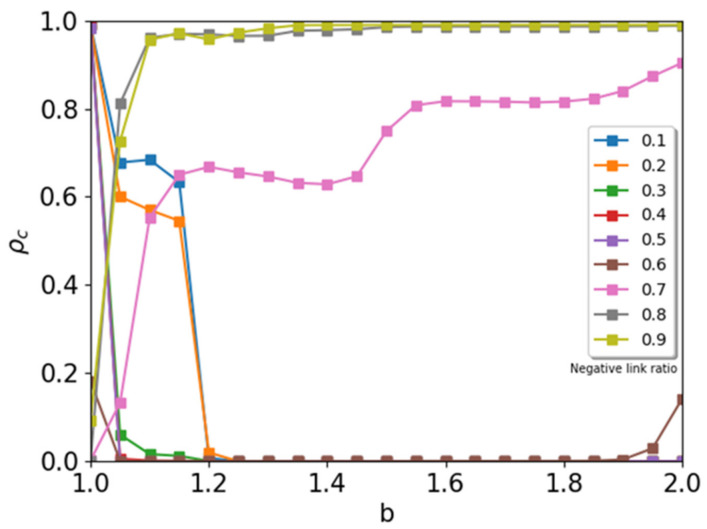
Ratio of cooperators as a function of payoff parameter b for a fixed ratio of negative links. When the ratio of the negative links is less than 0.3, the dynamics are similar to those of the original PD game. Around $r_{-}=0.6$, we observe a reentrant phase of cooperators at the high value of b>1.9. At $r_{-}>0.7$, the system shows a cooperating phase, regardless of temptation parameter b.

Next, we simulated the prisoner’s dilemma game on a signed network (square lattice with eight neighbors). We obtained order parameter $\rho_{c}$ as a function of payoff parameter b at a fixed fraction of negative links, $r_{-}$ (Figure 3). At a low value for negative links, $r_{-}$ < 0.3, we observed behavior similar to that seen in [20]. In this case, cooperators survive permanently below the critical value of the payoff parameter, $b<b_{c}\left(r_{-}\right)$. When the defector obtains a high payoff, the whole system is occupied by defectors at $b>b_{c}\left(r_{-}\right)$ because all players adapt to the defectors. We observe the typical shoulder of the order parameter below critical value $b_{c}\left(r_{-}\right)$. The value of the order parameter at the shoulder decreases when we increase the negative links. We increase the number of negative links further, and the shoulder disappears. The order parameter rapidly reaches the absorbing value $\rho_{c}=0$. The critical value, $b_{c}\left(r_{-}\right)$, decreases as the ratio of negative links increases. In this range, the competition between cooperators and defectors is severe. The defectors win above the critical value of b.

We observe an interesting behavior in cooperators when the ratio of negative links $r_{-}$ is greater than 0.5. At $r_{-}=0.6$, as shown in Figure 3, we observed a reentrant phase of cooperation. When payoff parameter b is increasing, we observe a coexisting phase (C1) below critical value $b_{c1}\left(r_{-}=0.6\right)\sim 1.04$. In this phase, the cooperators survive in a stationary state. Above critical value $b_{c1}\left(r_{-}=0.6\right)$, the whole system is occupied by the defector’s phase (D). In this phase, defection is a winning strategy. When we increase payoff parameter b further, we observe a new reentrant phase to the coexisting phase (C2) above $b_{c2}\left(r_{-}=0.6\right)\sim 1.9$. In the PD game with a signed network, the players with the negative sign follow a different payoff matrix, compared to the prisoner’s dilemma game with positive links. For agents connected by a negative link, the cooperator receives a greater benefit than a defector because the payoff for cooperators is −1. However, the payoff for defectors is −b. Therefore, at a high value for b, the system is in a mixed state with cooperators and defectors. In this situation, cooperators have enough of a benefit to obtain the payoff, and a portion of the cooperators survive. The cooperators survive in the boundary between negative and positive links.

When we further increase the ratio of negative links in the system, we observe a new transition from the defecting phase at b=1 to a coexisting phase between cooperators and defectors. For $r_{-}\;\geq 0.7$, there is a defecting phase at b=1. The system is in a stationary state with mixed agents (cooperators and defectors). At $r_{-}=0.7$, we observe two plateaus for the order parameter when we increase payoff parameter b. These plateaus are due to the competition of the players for the payoffs between positive links and negative links. At $r_{-}>0.8$, the system is mixed (cooperators and defectors). When we increase payoff parameter b, the cooperators survive in a steady state. The fraction of cooperators increases rapidly and reaches a stationary value. However, the order parameter is always less than 1. Cooperators and defectors mix. When the payoff parameter is increasing, the fraction of cooperators increases and approaches 1 ($\rho_{c}\to 1$) at a large value for b.

**Figure 4:**
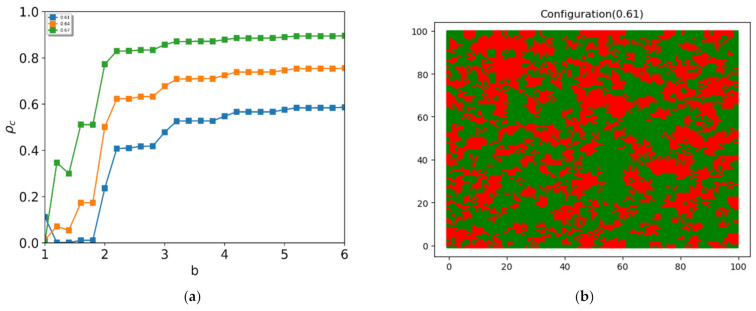
Order parameter and snapshot of the configuration on the game: (**a**) the order parameter versus control parameter *b* for $r_{-\;}=0.61$ (blue), 0.64 (orange), and 0.67 (green); (**b**) a snapshot of the configuration in the steady state at $r_{-}$ = 0.61 (green: cooperators, red: defectors). It shows a configuration for the mixture of cooperators and defectors at $r_{-}$ = 0.61.

To investigate the reentrant phase transition in detail, we measured the fraction of cooperators as a function of the payoff parameter up to *b* = 6 around the ratio of negative links at $r_{-}=0.6$. As shown in Figure 4a, the reentrant phase is observed near $r_{-}=0.6$. However, when the ratio of negative links, $r_{-}$, is greater than 0.64, the order parameter no longer shows the reentrant phase. The order parameter shows a zig-zag pattern at low values of *b* and saturates at a stationary value. As shown in Figure 4b, we observed dynamic oscillation in the defectors (shown in red) around the cluster of negative links. The cooperator and the defector coexist in a mixed state. In this mixed phase, the clusters of defectors survive permanently. The change in state occurs on the boundary of the cluster of defectors.

**Figure 5:**
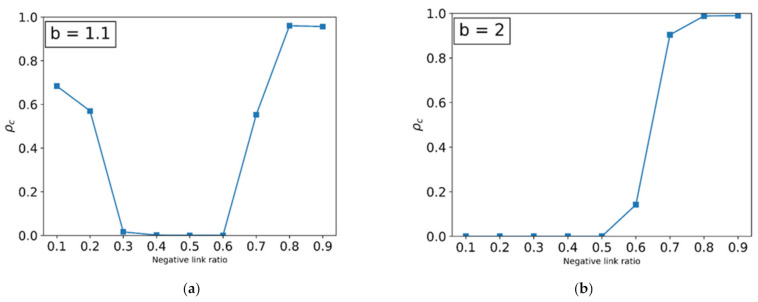
The fraction of cooperators versus the ratio of negative links for a fixed payoff parameter.

In Figure 5, we represent the fraction of cooperators as a function of negative links $r_{-}$ at a fixed payoff value for *b*. When the temptation parameter is close to 1 (b=1.1 in Figure 5a), the cooperating strategy can survive in C clusters as in the original Nowak and May [3] simple PD game. In addition, the fraction of cooperators, $\rho_{c}$, is greater than zero for the ratio of negative links up to $r_{-}=0.3$. In the range $0.3<r_{-}<0.6$, the order parameter is zero. As the ratio of negative links increases, D clusters become larger because defectors want to meet other defectors through the negative link (better loss avoiding). At $r_{-}>0.6$, the fraction of cooperators is non-zero because cooperation is a better strategy to avoid loss through the negative link. The system reveals the reentrant phase transition for the ratio of negative links. When we increase the negative links, the system shows a coexisting phase, an absorbing phase (only defectors), and a coexisting phase again. However, as the temptation parameter increases (for example, b=2 in Figure 5), the system shows a transition from an absorbing phase to a coexisting phase when we increase the ratio of negative links. When *b* ≫ 1, a cooperating strategy is better than defecting to avoid loss through the negative link. Therefore, the fraction of cooperators increases when the ratio of negative links increases.

## 4. Conclusions

We consider the prisoner’s dilemma game with a signed network. Two players with a negative link use a different payoff matrix, compared to the original payoff matrix of the prisoner’s dilemma game. The dynamics are controlled by temptation payoff *b*. At a low fraction of negative links, the dynamics are similar to those of the original PD game. The system shows a coexisting phase for cooperators and defectors at a low temptation parameter but, at a high temptation parameter, shows an absorbing state occupied by only defectors. In contrast, when the negative link density is high, the cooperator density becomes almost 1 as *b* increases. This is because players with a negative link suffer more payoff damage if they do not cooperate with each other. The negative link forces players to cooperate, so cooperating behavior is enhanced. We obtained the results of the mean-field theory with the signed network. The dynamics heavily deviate from the mean-field results. We obtained the phase diagram from a Monte Carlo simulation, and observed a reentrant phase at a particular value of the temptation payoff. The signed networks showed fruitful dynamics in the games.

The mixture game on the signed network can be applied to social dynamics when two choosing strategies are selected. One is the prisoner’s dilemma game and the other is the reverse prisoner’s dilemma game. The cases applying the prisoner’s dilemma game are well known. However, the cases applying the reverse prisoner’s dilemma game are new. In the reverse PD game, the CD or DD pairs retain their opinion even if the partner of the player defects. This is similar to the situation in which the status of political opinion is maintained. When we compare the CC and DC pairs, the cooperating pair is more beneficial than the DC pair. The mixed game we propose has some limitations because we apply the game to quenched signed networks and a square lattice with eight nearest neighbors. The player on the lattice site can change their status due to the influence of neighboring opinions. We can extend this mixed game into the annealed case, in which the sign of the link can change according to some rules of the dynamics. We can also apply the mixed game to the complex networks, such as small-world networks or scale-free networks. These will be topics for our future works.

## Figures and Tables

**Table 1:** Payoff matrix of the prisoner’s dilemma game. A defector obtains a bigger payoff than a cooperator (*b* > 1).

Strategy	Cooperation (C)	Defection (D)
Cooperation (C)	1	0
Defection (D)	b	0

## Data Availability

Data is contained within the article.

## Appendix A

Mean-Field Theory of the Prisoner’s Dilemma Game on a Signed Network.

Consider the mean-field theory of the PD game on the signed network. Let the state of the cooperator and the defector be a two-dimensional unit vector, as follows:

(A1) $$C=\left(\begin{array}{c} 1\\ 0 \end{array}\right)\;\&\;D=\left(\begin{array}{c} 0\\ 1 \end{array}\right)$$

The state of the player at site i is denoted $s_{i}$ = *C* (or *D*). Then, the total payoff, $U_{i}$, of player *i* is given by:

(A2) $$U_{i}=\sum_{\delta_{+}}s_{i}^{†}A\cdot s_{\delta_{+}}-\sum_{\delta_{-}}s_{i}^{†}A\cdot s_{\delta_{-}}$$

where $\delta_{+}$ denotes the nearest neighbors connected by a positive link, and $\delta_{-}$ denotes nearest neighbors connected by a negative link. The total number in the coordination for player *i* is $z=\delta_{+}+\delta_{-}=8$. The payoff matrix is given by:

(A3) $$A=\left(\begin{array}{cc} 0 & b\\ 0 & 1 \end{array}\right)$$

The average payoff for *C* and *D* strategies is given by:

(A4) $$U_{C}=z\rho_{c}\left(r_{+}-r_{-}\right)$$

(A5) $$U_{D}=z\rho_{c}b\left(r_{+}-r_{-}\right)$$

where $\rho_{c}$ is the density of the cooperators and the ratio of the negative links is given by:

(A6) $$r_{-}=\frac{N_{-}}{N_{+}+N_{-}}$$

and the ratio of the positive links is given by:

(A7) $$r_{+}=\frac{N_{+}}{N_{+}+N_{-}}$$

The transition probability from the initial state to the final state is given by:

(A8) $$W\left(D\leftarrow C\right)=\frac{1}{1+\exp\left(-\frac{\left(U_{C}-U_{D}\right)}{k}\right)\;}$$

(A9) $$W\left(C\leftarrow D\right)=\frac{1}{1+\exp\left(-\frac{\left(U_{D}-U_{C}\right)}{k}\right)\;}$$

We obtain the rate equation for the density of cooperators as follows:

(A10) $$\frac{\partial \rho_{c}}{\partial t}=\rho_{c}\left(1-\rho_{c}\right)\left[W\left(D\leftarrow C\right)-W\left(C\leftarrow D\right)\right]=-\rho_{c}\left(1-\rho_{c}\right)\tanh\left(\frac{U_{D}-U_{C}}{2k}\right)=-\rho_{c}\left(1-\rho_{c}\right)\tanh\left(\frac{z\rho_{c}\left(r_{+}-r_{-}\right)}{2k}\left(b-1\right)\right)\;$$
